# Is there a better future of healthy aging?

**DOI:** 10.3325/cmj.2020.61.75

**Published:** 2020-04

**Authors:** Mirjana Kujundžić Tiljak, Željko Reiner, Marijan Klarica

**Affiliations:** 1 Andrija Štampar School of Public Health, University of Zagreb School of Medicine, Zagreb, Croatia *mirjana.kujundzic-tiljak@snz.hr*; 2 Department of Internal Medicine, University Hospital Center Zagreb, University of Zagreb School of Medicine, Zagreb, Croatia; 3 Department of Pharmacology and Croatian Institute of Brain Research, University of Zagreb School of Medicine, Zagreb, Croatia

Population aging affects almost every sphere of society. The number of people aged 60 or older has surpassed 1 billion, and the majority of them lives in low-income and middle-income countries, often without access to basic resources and facing barriers to full social participation. The number of European citizens aged over 65 in the next 50 years will increase two times, while the number of octogenarians will increase three times. However, a longer life does not necessarily mean a healthier or a more active life. Today, unhealthy life years amount to almost 20% of a person's life.

An aging population, mainly due to the presence of chronic diseases, requires greater use of health care services, leading to unsustainable health care costs (1). The challenges posed by aging population in Europe have been the focal point of debates on the future of European Union. An increasing number of retired people, coupled with a shrinking working-age population, is expected to place additional burden on European welfare systems. European 2020 Strategy for Smart, Sustainable, and Inclusive Growth has been implemented with an aim to promote active aging policies. Besides eradicating poverty and social exclusion, the strategy emphasizes the need for “*promoting a healthy and active ageing population to allow for social cohesion and higher productivity*” (2). Similarly, the initiative of European Innovation Partnership in Active and Healthy Aging by the European Commission fosters innovation and digital transformation in active and healthy aging (3).

The WHO Decade of Healthy Aging (2020-2030) brings together multiple stakeholders, from governments and the public sector to the media and civil society, with an aim of improving the lives of older people (4).

The future of aging is being problematized based on the multidimensional concept of healthy aging defined as “*the process of developing and maintaining the functional ability,*” which enables well-being in older age. Older people have more complex health needs and are susceptible to developing chronic diseases. Frailty, a common clinical syndrome in older adults, marked by a greater risk for poor health outcomes, including incident disability and higher hospitalization and mortality rate, severely affects health care services and society as a whole (5).

Most health services treat acute conditions and manage health issues in a fragmented manner. Such fragmentation, as well as suboptimal time-management, could be especially dangerous for organizing adequate care for the elderly. There is a need for a health system reform that would ensure that each and every older person has access to evidence-based medical interventions and timely organized shared care. This is the only way to prevent further health deterioration, disability, and complicated care dependency later in life.

Active and healthy aging is a common problem in all European countries but could also be an opportunity for Europe to spearhead the quest for innovative solutions. Aging is one of the crucial societal challenges in Croatia, as reflected in the 2020 Croatian Presidency of the Council of the EU priority of the Ministry of Health of the Republic of Croatia – lifelong health care with the emphasis on challenges of aging.

**Figure 1:**
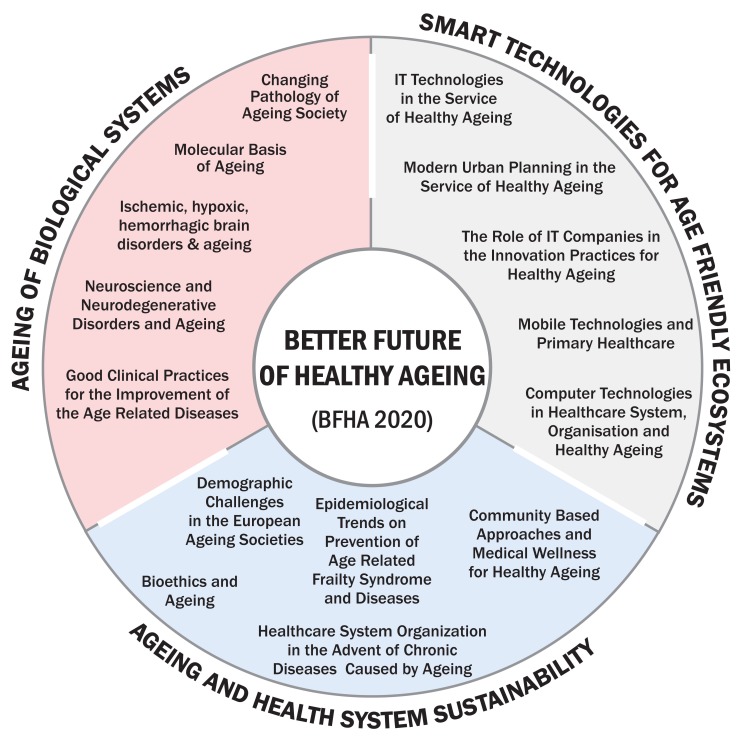
Better Future of Healthy Aging 2020 Conference topics

This theme issue of the *Croatian Medical Journal* is dedicated to the Conference on Better Future of Healthy Aging 2020 (BFHA 2020) (Figure 1), organized by the University of Zagreb School of Medicine in cooperation with the Ministry of Science and Education of the Republic of Croatia, under the auspices of the European Commission as part of the Croatian Presidency of the Council of the European Union (the Conference is organized with the contribution of all Croatian medical schools – University of Rijeka Faculty of Medicine, University of Split School of Medicine, and Josip Juraj Strossmayer University of Osijek Faculty of Medicine, as well as other faculties of the University of Zagreb – the Faculty of Electrical Engineering and Computing, Faculty of Economics and Business, and Faculty of Croatian Studies. Organizational support is provided by the Ministry of Health, Ministry for Demography, Family, Youth and Social Policy and Ministry of Foreign and European Affairs of the Republic of Croatia).

The Conference aims to promote research and innovations that improve health and well-being of the aging population, as well as to initiate discussion on transformation of health and care services into more digitalized, long-term, integrated, and personalized care models, while promoting innovative ecosystems in order to deliver a better quality of life among the elderly.

The first objective of the Conference is to address the issues of aging of biological systems and present the state of the art in the areas of regenerative medicine, neuroscience, clinical medicine, and other fields of medicine. The accent is on personalized and integrated medicine and innovative translational research leading to promising applications in regenerative medicine. The conference will also include discussions on advanced personalized diagnostic strategies enabling individualized therapy and accurate predictions of treatment outcomes. The implementation of modern sophisticated genomic methods in routine diagnostics for personalized medicine and the scale up of demand-driven innovations in health care systems includes organizational, economical, technical, and clinical aspects. If we want to extend healthy and independent living, we need to create robust and sustainable solutions potentially applicable in any EU state.

The second objective of the conference is to assess the use of smart technologies in age-friendly ecosystems. The conference aims to initiate a discussion on the possibilities to scale up innovations and solutions for age-friendly environments, applications for independent living, and solutions for age-friendly buildings, cities, and environments, undertaken by different European cities, regions, or municipalities. Smart ICT solutions and advanced artificial intelligence (AI) can be used to provide personalized health care and social services, overcome immobility, cognitive, and vision problems, and improve general quality of life. Digital technologies can encourage all groups of patients, and the elderly in particular, to assume a more active role in their health management. Hopefully, the conference discussions will lead to discovering the ways how all European citizens can meaningfully use these new technologies and benefit from them.

The third objective is to analyze the issues of aging and health care system sustainability at various levels – institutional, regional, state, and EU level. In the area of health systems sustainability, the conference focuses on the following themes and their relation to extended life and aging: health system financing, health system organization and structuring bottom up policy with successful examples, and demographical and ethical challenges. The role of digitalization in health care is vital in advancing solutions to challenges related to all three conference topics, particularly as health systems are often not keeping pace with the integration of the new technologies. The possible solutions to overcome disparities in the availability of technological developments and health and digital literacy of the elderly in different European countries and regions are particularly important. The BFHA Conference aims to create a platform for sharing the successful examples of using advanced technologies to increase the functionality of aging citizens and for learning from these examples.

Addressing the use of advanced technologies for improving the functionality and well-being of aging citizens to the benefit of a transformative and mission-oriented European research and innovation agenda goes beyond the traditional focus on scientific impacts of research. It emphasizes societal impacts, structuring impacts on policymaking and policies, as well as impacts on innovation and economy.

Unfortunately, at the beginning of WHO Decade of Healthy Aging (2020-2030) the whole world is facing the coronavirus disease (COVID-19) pandemic, which endangers mostly elderly and chronic patients. The pandemic outlines the importance of disease prevention using well known hygiene measures. However, it also reveals the unequal access to health services faced by older patients. In some countries, top ranking hospitals recommended that patients with COVID-19 who are older than 80, between 70 and 80 years with one-organ failure, and between 60 and 70 years with two-organ failure should not be given priority if there is a lack of intensive care units beds.

Croatian bases its disease prevention and health promotion strategies on the ideas of Dr Andrija Štampar, one of the founders of the WHO (6). He began his fight for better public health in the first half of the 20th century (7). At that time, sanitary and hygiene situation was poor, and the major health problem were infectious diseases, particularly malaria and tuberculosis (8). Andrija Štampar became highly active in public health efforts in Croatia and abroad. His definition of “*health as a state of complete physical, mental and social well-being and not merely the absence of disease or infirmity”* is still a part of the WHO Constitution, which was adopted on April 7, 1948. In his speech as Acting President of the First world Health Assembly held in Geneva in 1947, Štampar said the following (8): “*Disease is not brought about only by physical and biological factors. Economic and social factors play an increasingly important part in sanitary matters, which must be tackled not only from the technical, but also from the sociological point of view.... Health should be a factor in the creation of a better and happier life. Since health for everyone is a fundamental human right, the community should be obliged to afford all its people’s health protection as complete as possible. Medical science must adopt a positive rather than defensive attitude. Great tasks await the World Health Organization in this field, and its future success will largely depend on its ability to put these ideas into practice*.”

In the light of his words, one can conclude that all people, regardless of their origin, should be granted the opportunity to live a long and healthy life. Yet, the environments in which we live can promote health or degrade it. Environment and spatial epidemiology play an important role in defining the health risks of a population. Another important factor is the accessibility to quality health regardless of social status and other sources of deprivation. Population aging makes these issues increasingly more visible. Healthy aging is about creating the social climate and opportunities that enable people to thrive throughout their lives.

The COVID-19 pandemic has shown the importance of every person’s involvement in creating a healthy environment, healthy relationships, building solidarity, and social awareness of health needs. Let us use this newly obtained attitude to improve the care for the elderly and lead the world to a better future of aging.
